# Language and the Moving Body: Directive Actions With the Finnish *kato* “look” in Nature-Related Activities

**DOI:** 10.3389/fpsyg.2021.661784

**Published:** 2021-06-04

**Authors:** Pauliina Siitonen, Mirka Rauniomaa, Tiina Keisanen

**Affiliations:** Languages and Literature, Faculty of Humanities, University of Oulu, Oulu, Finland

**Keywords:** directive, imperative, *kato* “look”, noticing, prompt, showing, verbs of perception

## Abstract

The article explores how social interaction is accomplished through intertwined verbal and bodily conduct, focusing on directive actions that include a second-person imperative form of the Finnish verb *katsoa* “to look,” typically *kato*. The study draws on video recordings of various outdoor activities in nature, mostly from family interaction with small children, and employs interactional linguistics and conversation analysis as its analytic framework. The directive *kato* actions in focus are produced (1) as noticings, to initiate a new course of action by directing the recipient to look at and possibly talk about a target that the speaker treats as newsworthy; (2) as showings, to initiate an evaluative course of action by directing the recipient to look at and align with the speaker’s stance toward the target; or (3) as prompts, to contribute to an ongoing course of action by directing the recipient to do something relevant to or with the target. Apart from the use of *kato*, the actions differ in their design. In noticings, the target is typically named verbally and pointed at through embodied means, but the participants remain at some distance from it (e.g., *kato muurahaispesä tuossa* “look an anthill there”). In showings, the participant producing the action typically approaches the recipient with the target in hand, so that the naming of the target is not necessary but, by evaluating the target themselves, the shower explicates how the target should be seen (e.g., *kato kuinka jättejä* “look how giant {ones}”). In prompts, neither the target nor the intended action is named, but the target is typically indicated by embodied means, for example, by the participants’ approaching and pointing at it, and the intended action is inferable from the participants’ prior conduct (e.g., *kato tuossa* “look there” and pointing at a berry in the participants’ vicinity when berry picking has been established as relevant). By examining these three grammar-body assemblages, the article uncovers regularities in the co-occurrence of multiple modalities and contributes to new understandings of language use in its natural ecology – in co-present social interaction.

## Introduction

A common problem in the midst of our everyday activities is how to get others to do something. Directive actions are one central means in this, and, as such, have also received a sustained amount of attention in language studies. The classic definition of directives in spoken interaction considers them as actions that aim to control the recipient’s conduct in some way (Ervin-Tripp, 1981, p. 196). Various directive actions, such as requests, advice giving, proposals, and instructions, have provided a fruitful arena for social-interactional research to explore how collaboration, assistance, or resistance is enacted in directive sequences (see, e.g., Kendrick and Drew, 2016; Sorjonen et al., 2017). On the other hand, others have proceeded to explore the ways in which participants “mobilize” action from others (Taleghani-Nikazm et al., 2020). Such a perspective highlights the role of the activities and the trajectories of action involved in making particular kinds of responses or actions by co-participants relevant (see also Stivers and Rossano, 2010). This article joins in the discussion by showing how specific embodied practices, participants’ movement, and their relative proximity with reference to each other and the object of their joint attention, created by a directive action, combine in systematic ways with linguistic turn design. Drawing on face-to-face interactions among speakers of Finnish during various outdoor activities in nature (mostly from families with small children foraging or trekking), the article presents three different grammar-body assemblages of how participants guide others to act, or not to act, in a specific way with directive actions.^1^

At focus here are directive actions that include a second-person imperative form of the Finnish verb *katsoa* “to look.” The most frequent form in the data is the colloquial second-person singular *kato* “look.” Hakulinen and Seppänen (1992) have noted that in addition to functioning as an imperative verb that has the concrete meaning of “looking,” *kato* functions as a particle that is used as an attention getter or an explanatory connective, that is, it may be used to signal the point in a telling and to mark an explanation (see also Siitonen et al., 2019). Indeed, in Finnish and several other languages, some verbs of (visual or auditory) perception have conventionalized into discourse markers that are used as resources for regulating interaction and managing interpersonal relations: such discourse markers are employed, for example, as a resource for directing the recipient’s attention to some abstract content, disaligning with a prior turn, launching or redirecting a course of action, or making claims of evidential vindication (see Hakulinen and Seppänen, 1992; Keevallik, 2003, p. 203–204, 207, 214; Sidnell, 2007; Kendrick, 2019). In this study, however, we draw on social activities in which participants move and act in a material world populated with physical objects and orient primarily toward features of their environment. Our focus is on *kato* actions with which the participants direct each other’s embodied conduct as is essential here and now in terms of an incipient or ongoing activity. Such *kato* actions constitute the clear majority of the *kato* turns in our data from natural settings.

**Figure 1:**
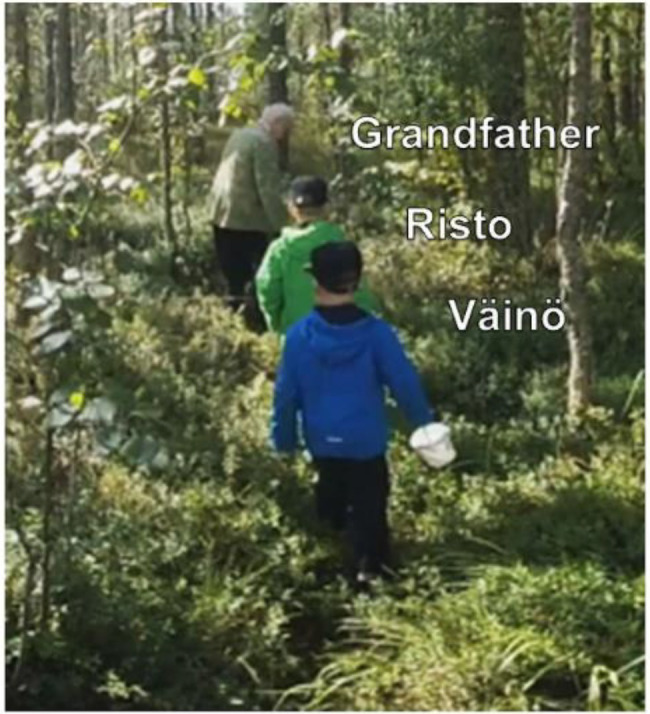
Grandfather, Risto and Väinö are walking into the woods to pick bilberries.

Let us briefly discuss an example from the data to show how central the participants’ embodied conduct is in designing and responding to the *kato* actions under examination. In Example 1, grandfather, Väinö (5 years), and Risto (7 years) are walking into the woods to pick bilberries (Figure 1). During this brief extract, the participants produce three directive actions that include the verb *katsoa* “to look” in the second-person imperative (lines 2, 13, and 15).

**Example 1:**
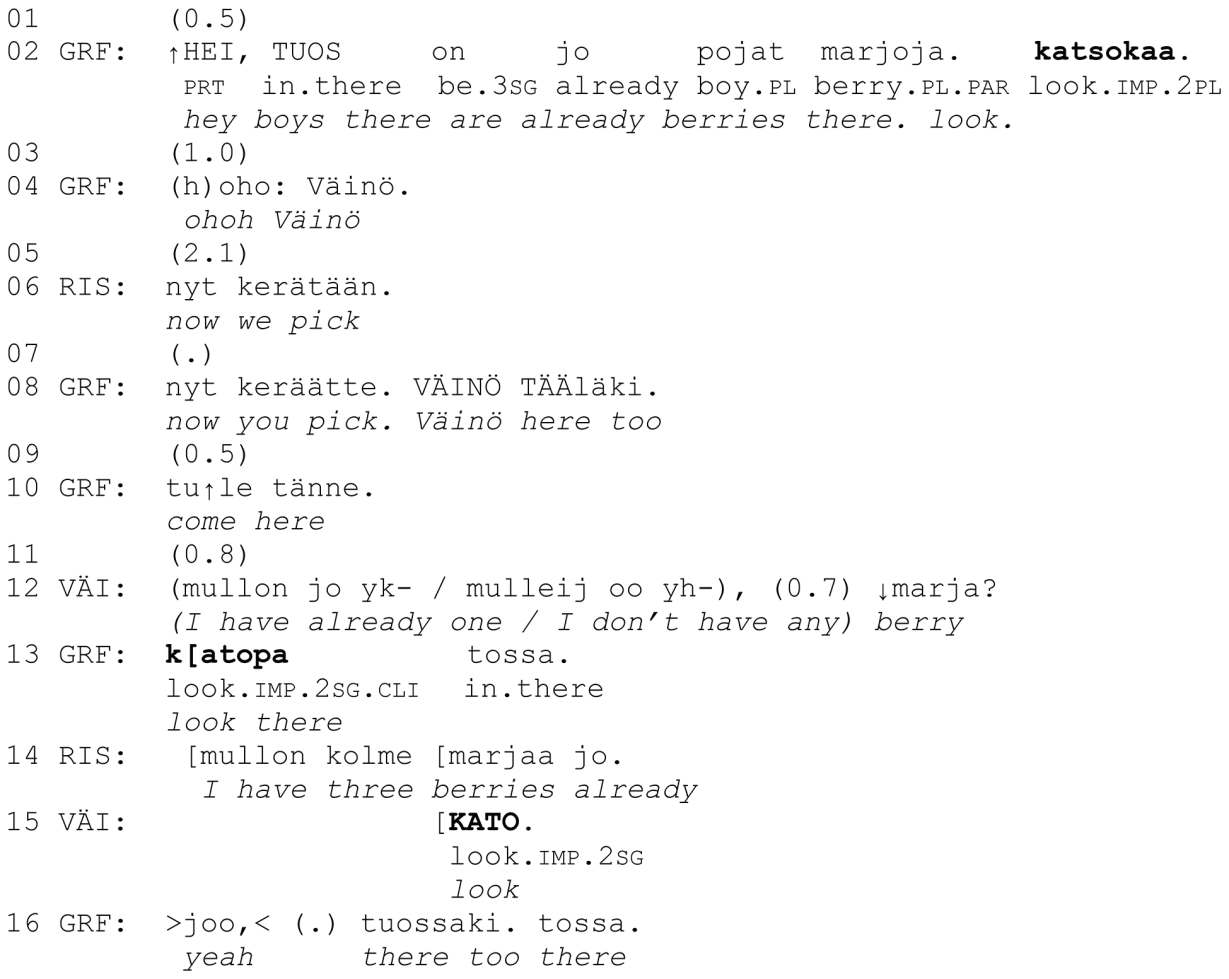
51HANS mustikassa 1 (00:01:14).

Focusing first on the linguistic design of the turns, we can analyze them as follows: *katsokaa* “look” as a second-person plural imperative (line 2), *katopa tossa* “look there” as a cliticized second-person singular imperative followed by a deictic term (line 13), and *kato* “look” as a second-person singular imperative (line 15). As such, the three turns can already be understood to direct the recipients’ attention to an object that is visually accessible to both the speaker and the recipient(s). Taking then the details of both sequential and spatial context into account (albeit we do not provide the multimodal transcript of the example at this point), we begin to see that each of the focus turns constitutes a part of a particular social action. *Katsokaa* “look” (line 2) is incrementally added to a turn that also includes an attention-getting “hey,” the summons “boys” and an initial identification of a location where berries can be found. Moreover, it is produced when the participants, Väinö and Risto especially, are close enough to see, if not yet pick, the berries. *Katsokaa* “look” can, thus, here be considered as part of a *noticing*. *Katopa tossa* “look there” (line 13), in turn, is produced when berry-picking has already been established as the participants’ ongoing joint activity, and it is addressed to Väinö, who stands close to grandfather as well as the berries and who has indicated that he has not yet picked many berries (line 12). Here, grandfather’s *katopa tossa* “look there” works in *prompting* Väinö to pick the berries pointed out to him. Finally, *kato* “look” (line 15) is produced by Väinö as he brings his berry container to grandfather’s line of vision, *showing* grandfather its scarce contents.

In what follows, we first discuss previous literature on the actions of noticing, showing, and prompting and briefly introduce our research materials and methods. In section “Analysis and Findings,” we provide detailed analyses of our data. We will begin with *kato* noticings, which are used to initiate a new course of action by directing the recipient to look at and possibly talk about a target that the speaker treats as newsworthy. After that we will discuss *kato* showings, which direct the recipient to look at and align with the speaker’s stance toward the target. Finally, we will show how *kato* prompts contribute to an ongoing course of action by directing the recipient to do something relevant to or with the target. In section “Directive *kato* Actions as Grammar-Body Assemblages,” we summarize and expand on our observations on the different directive *kato* actions by considering their embodied design and by presenting two linguistic turn design practices used in these actions. We conclude by arguing that the three different *kato* actions are identifiable as noticings, showings, and prompts, respectively, only if we take into consideration their overall linguistic and embodied design as well as the sequential and spatial positions in which they are produced. While establishing or maintaining some specific target as the focus of the participants’ joint attention, the three *kato* actions entail increasing multimodal and multisensorial involvement from the recipient.

## Previous Research on Noticings, Showings, and Prompts

Previous interactional research on “noticings” has viewed them as being preceded by a perceptual observation or a cognitive change in the speaker that their noticing then embodies (Heritage, 2005, p. 188; Schegloff, 2007: footnote 17). Such actions thus form one convenient means in social interaction for mobilizing the recipient’s attention on some event or feature in the immediate surroundings or prior talk (Schegloff, 2007, p. 219) and thereby establishing joint attention (Tomasello, 1995, p. 106–107). Whether under the label of noticings or some other related terms, studies have explored how participants negotiate the meaning of and their relative position toward noticed referents (Goodwin and Goodwin, 2012) including cases in which one participant is regarded as responsible for them (as in one’s clothing or home; Pillet-Shore, 2020). Previous studies on noticings have also explored how states of incipient talk develop into sequences of focused interaction through comments on the physical surroundings (Keevallik, 2018), in response to some sudden problematic event (Keisanen, 2012) or not, or how the organization of multiple ongoing activities may be managed with noticing-launched interventions (Helisten, 2019). In many cases, studies have shown how the vocal and embodied conduct of the participants contribute to the locally occasioned meanings and purpose of doing noticing (e.g., Kääntä, 2014; Laanesoo and Keevallik, 2017). The current study contributes to the multimodal studies on joint attention and action by focusing on social actions in which the linguistic resource *kato* directs the recipient to look at something (see section “*Kato* noticings”). Not all “environmental” noticings (Sacks, 1992, p. 90), such as *mm it smells so good in here* or *your hair looks so cute* (Pillet-Shore, 2020, p. 2, 4) or *meil on vauhti pudonnu* “we have lost speed” (Rauniomaa et al., 2018, p. 6) are designed to do that, but they are implemented to accomplish other aims.

Another type of action that *kato* turns are used to implement include “showings” (see section “*Kato* showings”). A key study on showing sequences between children and caregivers laid out the basic order of actions in such sequences, proceeding from (1) a child showing an object in their hand to a recipient; (2) a response from the recipient, often identifying the object; and (3) the child then treating the response as adequate or not (Kidwell and Zimmerman, 2007, p. 593). In addition to the identification of the showed item, showing sequences may also be designed to involve the assessment or evaluation of the target of joint attention (Licoppe, 2017; Searles, 2018). Though this is not always the case, showing sequences involve rather frequently the shower’s direct involvement with objects, be it smartphones (e.g., Weilenmann and Larsson, 2001; Aaltonen et al., 2014; Avgustis and Oloff, submitted), clothing (Fasulo and Monzoni, 2009; Licoppe and Tuncer, 2019), or other relevant items (Gerhardt, 2019). In remote mediated interactions, which provide one perspicuous context for showing sequences in adult interaction, the shower may also enact gestural showings (Licoppe, 2017; Due and Lange, 2020), turn the computer to show the environment for the recipient (Zouinar and Velkovska, 2017) or engage in entirely digital showings by sharing their screen or a link to some relevant materials (Rosenbaun and Licoppe, 2017). Of special interest in terms of the present study is the finding of Licoppe and Tuncer (2019) on video-mediated interactions in French, in which showing actions are in two-thirds of the cases prefaced with the directive *regarde* “look.” As will be discussed in section “Analysis and Findings,” it is the embodied, and material environment that provides the resources for the participants to collaboratively design their actions as *kato* showings or *kato* noticings, both of which centrally involve the invoking of joint attention for joint action.

“Prompts,” in turn, may be employed to encourage the recipient to elaborate their previous actions in different ways. In one of the first studies to use the term prompt and to explore how such actions work as other-initiated repair, Lerner (2004b) identifies a linguistic practice by which speakers can prompt another to add an increment to their prior turn, and thereby extend their earlier contribution. The linguistic items of English discussed include, for example, *to*, *for*, *rather than*, and *meaning*. Relatedly, Raymond (2004) discusses how the English stand-alone *so* may be employed in managing the ongoing course of action to prompt the previous speaker to elaborate on the import of their prior turn or action. The use of linguistic resources for eliciting more talk from the recipient has also been discussed, for instance, in the context of psychotherapy (Muntigl and Hadic Zabala, 2008) and as regards how teachers in a classroom setting can support children in their word searches (Radford, 2010). In addition to verbal prompting, Radford (2010) discusses embodied prompts, which do not give a verbal model for the child on what the searched for word is but utilize gaze and gesture instead. As mentioned above, prompting may be used to manage the sequential organization of the ongoing action. This aspect is taken up in Kamunen and Haddington (2020) as they examine activity transitions from a current activity to some other imminent activity. Explicit prompts are discussed as the means to accomplish an immediate but coordinated transition to the new activity: these include embodied prompts, such as nods and gestures, and verbal prompting turns, such as *we will now change the sample* or *I think we can start* (Kamunen and Haddington, 2020, p. 104). Here, we continue this line of research in studying how participants make relevant certain kinds of actions from others by producing *kato* prompts.

## Data and Methods

Our data come from various outdoor activities such as foraging, trekking, and orienteering. The participants are family members (children aged 2–7 years), groups of friends, or participants on organized outings, who have given their informed and voluntary consent before participating in the study. We have removed identifiable information from the transcripts by using pseudonyms for the participants and by retouching the frame grabs from the videos. The data, amounting to approximately 24 h, were recorded by researchers and/or participants with one to three handheld or head-, chest-, or tripod-mounted cameras; the most recent piece of data (approximately 15 min) was recorded with a handheld 360-degree camera.

The data include 279 turns in which the speaker uses some form – standard or colloquial, singular or plural, and cliticized or non-cliticized – of the second-person imperative of the verb *katsoa* “to look,” most typically the colloquial singular non-cliticized form *kato* “look.” The distribution of the different uses of *kato* actions in our data is presented in Table 1.

**Table 1:** *Kato* actions in the data.

*Kato* action	Example from data	*N*
1. *kato* directing to look and elaborate, or **noticing**	*kato muulahaitpetä* “look an anthill”	83
2. *kato* directing to look and assess, or **showing**	*äiti kato kuinka paljo meikä on saanu jo* “mother look how many {berries} I have already”	68
3. *kato* directing to look and do, or **prompt**	*katopa tossa* “look in.there” (as a response the recipient picks a berry)	92
4. *kato* as an attention getter or explanatory connective	*ei saa. kato sua ossuu silimään*. “do not do that. *kato* it will hit you in the eye”	31
5. *kato* as a token of general wondering	*kato. onko haaparousku* “*kato*/well well. is it a pickle milk-cap”	5
All		279

As we have shown earlier with a slightly smaller data set, the participants who are engaged in physical activities in natural settings most often produce *kato* turns as components of multimodal directive actions (Siitonen et al., 2019). In other words, multimodal *kato* actions direct the recipient to do something concrete in a material world: such actions either direct the recipient to turn their gaze to look at an object or feature of the surroundings (*kato* actions 1 and 2 in Table 1) or to carry out a bodily action that is somehow relevant in terms of an ongoing activity (*kato* action 3 in Table 1). In clearly fewer cases in our data, the linguistic item *kato* has lost its verb-like features and functions as an attention getter or explanatory connective (*kato* action 4 in Table 1) or as a token of general wondering (*kato* action 5 in Table 1). We have also pointed out that the proportion of cases in which *kato* can be considered as an imperative verb (*kato* actions 1–3) to cases in which *kato* is better understood as a particle (*kato* actions 4 and 5) may be different when the type of social activity is something else than physical activity in nature. Indeed, in the data presented by Hakulinen and Seppänen (1992), in which 4–5 friends or peers engage in spontaneous informal conversations or in conversations that are task-oriented but that do not require the participants to move, the proportion is quite the opposite. In their data, in only 7 out of some 200 cases, *kato* was used as an imperative verb; in the overwhelming majority of cases, *kato* was used as a particle (Hakulinen and Seppänen, 1992, footnote 1, p. 530, 532). The different distributions of the interactional functions of the *kato* turns in the data examined by Hakulinen and Seppänen (1992) and in the data analyzed by us thus highlight the fact that different social activities allow for or make relevant different social actions.

In this study, our focus is only on the *kato* actions that guide the recipients’ embodied conduct, directing them to look (*kato* actions 1 and 2) or to carry out some other bodily action in addition to looking (*kato* action 3, or *prompts*). Unlike in our earlier study, we now further divide the former into *noticings* and *showings* (Table 1). The *kato* actions in which *kato* is better understood as a particle (*kato* actions 4 and 5) are not analyzed here.

We approach language and social interaction from the perspectives of interactional linguistics (e.g., Couper-Kuhlen and Selting, 2001, 2018) and multimodal conversation analysis (Mondada, 2014) to examine what interactional functions are furthered by particular grammatical forms and how their use is intertwined with the employment of other interactional resources such as gaze, gestures, body movements, material objects, and space. We have transcribed the examples according to the basic conversation-analytic conventions (see Jefferson, 2004) and the conventions of Mondada (2019) for multimodal transcription and provided glosses for the focus turns. We analyze the sequential unfolding of naturally occurring interaction, identifying and explicating the practices through which conduct in social activity is produced and understood (e.g., Schegloff, 2007).

## Analysis and Findings

In this section, we first analyze actions with which the speaker directs the recipient to look at a concrete object in their physical environment, establishing joint attention to the object for the benefit of collaborative action. We call these actions either *kato* noticings or *kato* showings, depending on their multimodal formation and interpretation as part of the ongoing activity. We then move on to analyze actions that we call *kato* prompts and show that with these actions the speaker directs the recipient to carry out a bodily action that is somehow relevant in terms of an ongoing activity.

### *Kato* Noticings

When *kato* is used in a turn that directs the recipient to look at an object in the surrounds, the target of the looking is most often mentioned explicitly. In the majority of such cases, the target is uttered after the word *kato* even though there are cases in which the order of the constituents is the opposite (e.g., Example 1, line 2). The target may also be pointed at, but usually so that the participants remain at some distance from it. Although the target may have been available to the participants’ perception also previously, *kato* noticings can be seen as multimodally produced interactional noticings that, by registering the object (or some feature of it), make it relevant for further talk and action, which it has previously not been (on interactional noticings, see Schegloff, 2007, p. 87, footnote 17, p. 219). In this way, *kato* noticings usually initiate a new sequence.

Example 2 represents a case in which conventional linguistic means to produce a noticing, including *kato* “look,” are intertwined with gaze and body orientation toward the target in the environment (see Goodwin and Goodwin, 2012, p. 276). Here, 4-year-old Risto, 2-year-old Väinö, grandfather, mother, and father are picking berries in the woods (the last two are off camera). The example starts when Väinö is sitting on the ground, unable to get up without help, and grandfather is busy assisting him. In the middle of grandfather’s and mother’s reasoning about and laughing benevolently at Väinö’s trouble, Risto produces a noticing about an anthill (lines 4, 7, and 8), initiating his verbal turn with *kato* “look.”

**Example 2:**
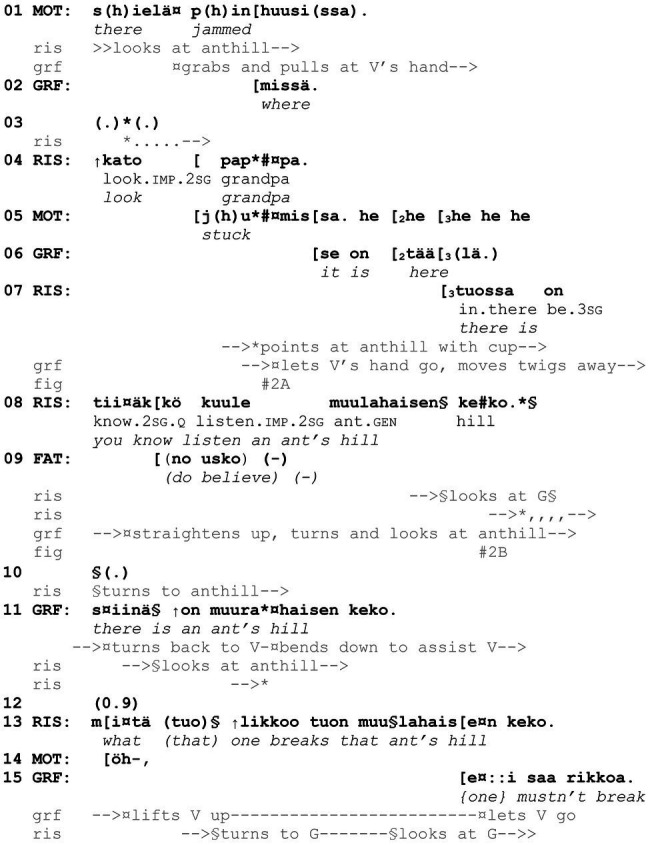
22 HANS Mustikassa II (00:07:37 / 00:7:15).

**Figure 2:**
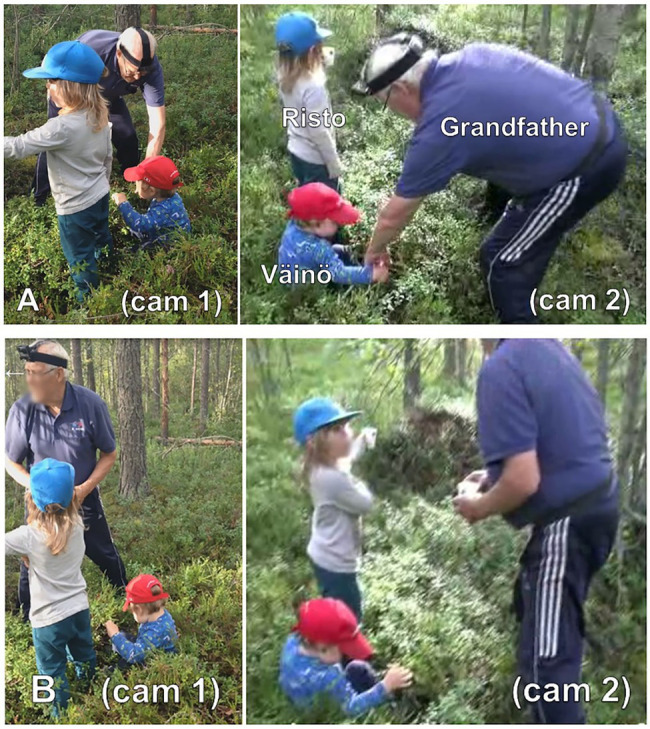
Risto looks at and points to the anthill but remains at some distance from it; grandfather assists Väinö in getting up **(A)**. Risto points to the anthill but looks at grandfather; grandfather looks at the anthill **(B)**.

In lines 1–6, mother and grandfather orient to Väinö, while Risto stands still with his back toward the others, looking at the anthill from a distance of a few meters. Halfway through the caregivers’ activity, that is, after grandfather’s turn *missä* “where” (line 2), with which he either asks the others where exactly Väinö has caught his feet or verbalizes his own search for the cause of the trouble, Risto initiates a new sequence. He summons grandfather “into a framework of collaborative mutual orientation” (Goodwin and Goodwin, 2012, p. 273) by directing him to look at the anthill, using embodied, spatial, and linguistic resources. More specifically, he stretches out his left arm to point at the anthill (with a paper cup in his hand) and produces the first part of the noticing, *kato pappa* “look grandpa,” as the pointing reaches its apex (lines 3–4, Figure 2A). At this moment, grandfather is still busy with his ongoing activity and does not look at Risto or in the direction that Risto is pointing to. Unaware of this, in overlap with the adults’ talk and laughter, Risto incrementally adds the next part of the noticing, *tuossa on tiiäkkö kuule muurahaisen keko*^2^ “there’s you know listen an ant’s hill” (lines 7–8). He uses several linguistic resources typical of constructing noticings: the imperative-formatted perceptual verb *kato* “look.imp.2sg,” the address term *pappa* “grandpa,” the deictic term *tuossa* “in.there” and the categorization *muulahaisen keko* “an ant’s hill” (on similar resources in English interaction, see Goodwin and Goodwin, 2012, p. 276; on the deictic term *tuossa*, see Laury, 1997, p. 74). Furthermore, he pursues an acknowledgement, a recognition of the target, with *tiiäkkö* “you know” (lit. “do you know,” see Suomalainen, 2020, p. 46–47) and marks the target as the high point of the turn with *kuule* “listen” (see Hakulinen et al., 2003, p. 208). At the end of his verbal noticing turn, Risto also glances at grandfather, thus ensuring that they share the same focus of attention (line 8, Figure 2B).

Grandfather interprets Risto’s multimodal action as a noticing that directs him to look at and acknowledge the nominated target: beginning in the middle of Risto’s *tiiäkkö* “you know,” grandfather straightens up and turns to look at the anthill (line 8, Figure 2B). Then, he verbally affirms Risto’s recognition of the target (*siinä on muurahaisen keko* “there is an ant’s hill,” line 11), albeit he already turns back to Väinö and bends down to assist him. At the same time, Risto looks at the anthill again and asks about the consequences of breaking the anthill [*mitä (tuo) likkoo tuon muulahaisen keko* “what (that) one breaks that ant’s hill,” line 13]. In so doing, he expands the noticing into a “knowledge exploration,” which is a means typically used by children to make imaginative inquiries about the world (Goodwin, 2007, p. 94; see also Waters and Bateman, 2015). It is worth noting that, despite their continuous orientation to the anthill, Risto and the caregivers do not approach the anthill at any point during the noticing sequence or the following knowledge exploration (for an analysis of the latter, see Rauniomaa et al., forthcoming 2021).

**Figure 3:**
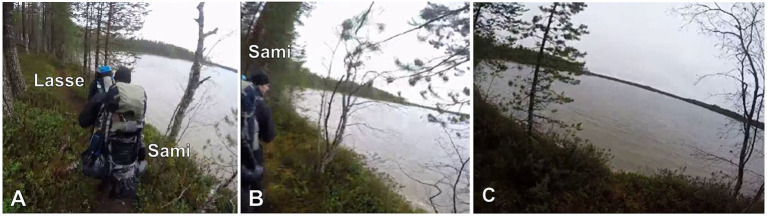
Lasse and Sami walk along a lakeside path; the head-mounted camera worn by Janne is directed toward them **(A)**. Sami looks at the lakeside; the head-mounted camera worn by Janne turns toward the lake **(B)**. The head-mounted camera worn by Janne is directed toward the lake(side) **(C)**.

Similar linguistic resources, typical of noticings, used in Example 2 are also employed in Example 3. Here, too, the target of the noticing (“lakeside,” line 5) has been available to the participants’ perception for some time already, but it has not been made the object of their joint attention earlier. In contrast with Example 2, however, here the noticing is not produced in the middle of a talk activity but after a lapse of 4.7 s (line 4) and in parallel with an ongoing walking activity. Example 3 involves four young men, Lasse, Sami, Janne, and Kalle, who are trekking in the woods, walking in single file along a lakeside path. Janne, the second to last in the file, wears a head-mounted camera, and hence the only participants on camera are Lasse and Sami, the first and second in the file, respectively. The extract starts when Sami asks about people whom they have seen earlier, possibly fishing on the lake (line 1). In response, Lasse accounts for not being able to answer the question and evaluates his own epistemic access to the topic, thus effectively closing the sequence (line 3, Figure 3A; see Schegloff, 2007, p. 188).

**Example 3:**
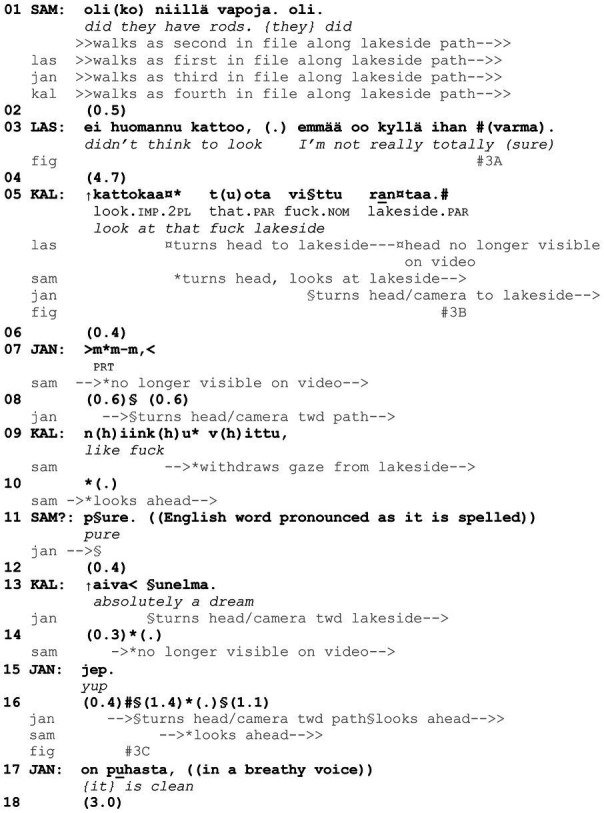
42COACT Salami buddy 6 (00:03:11).

At the beginning of the extract, talk about people on the lake possibly having rods fades out (lines 1–3) and a 4.7-s lapse ensues (line 4). The lapse ends as Kalle, who is off camera, initiates a new sequence with a *kato* turn that directs the others to view the scenery: *kattokaa tuota vittu rantaa* “look at that fuck lakeside” (line 5; on resolving lapses by registering some perceptible aspect of the situated environment, see Hoey, 2018, p. 339). In addition to using the second-person plural imperative verb *kattokaa* “look” that designates all the co-participants as recipients, Kalle uses the demonstrative pronoun *tuota* “that,” which locates the speaker outside the sphere of the referent (Etelämäki, 2009, p. 40) and categorizes the target with *rantaa* “lakeside.” Furthermore, he inserts the expletive *vittu* “fuck” in the middle of the NP *tuota rantaa* “that lakeside.” Since the expletive is not in the genitive case, it is not used as an attribute of the noun *ranta* “lakeside” (cf. *tuota vitun rantaa* “that fucking lakeside”; see Hakulinen et al., 2004, § 1726) but rather as an attention getter that expresses an affective stance and projects the high point to follow (see also Hakulinen et al., 2003, p. 208; Hakulinen et al., 2004, § 1727). In this way, the expletive also upgrades the action (see also Hoey et al., 2020). Nevertheless, Kalle does not make the valency of his affective stance explicit nor account for why he produced the expletive, that is, whether the lakeside is to be admired or to be shocked at (cf. Sacks, 1992, p. 495; see also Hakulinen and Sorjonen, 2012, p. 148; Avgustis and Oloff, submitted).

The recipients recognize Kalle’s turn as a noticing even before the verbal turn is brought to completion: they align themselves with it by turning their heads to the right (line 5, Figure 3B). However, the first verbal response to Kalle’s noticing is Janne’s minimal *mm-m*, which acknowledges the target (“that lakeside”) but displays neither admiration nor disapproval of it (line 7, see also Siitonen and Wahlberg, 2015, p. 78; Avgustis and Oloff, submitted). The participants keep on walking, and Janne starts to turn his head (and camera) in the direction of the path again (line 8). Kalle treats the others’ embodied and minimal verbal responses to the noticing as insufficient and initiates stance taking toward the target with the words *niinku vittu* “like fuck” (line 9) that projects a forthcoming assessment. However, he does not finish the utterance. Next, Sami expresses a candidate stance by building on Kalle’s initiation and collaboratively completing the assessment with the adjective *pure* (he pronounces the English word *pure* as it is spelled, line 11). The assessment is then confirmed and upgraded with *aivan unelma* “absolutely a dream” by Kalle (line 13; see Bolden, 2003; Lerner, 2004a). Halfway through Kalle’s verbal turn, Janne turns his head (and camera) toward the lake again (Figure 3C) and agrees with *jep* “yup” (line 15). Janne also displays his independent access to the target with *on puhasta* “{it} is clean” (line 17; on avoiding stance-taking before the first speaker’s stance is explicit, see also Avgustis and Oloff submitted). Throughout the noticing sequence and disambiguation of the appropriate stance about the scenery, the participants continue walking and slow down only slightly (walking speed not indicated in the transcript) as they turn their heads to look at the lakeside. By doing so, they display their understanding of the ongoing activity as looking at and elaborating on (in this case, assessing collaboratively) the nominated target from a distance, similarly to the participants in Example 2. Furthermore, Example 3 has shown that *kato* noticing sequences may include evaluation. By contrast, evaluation is in effect an essential component and salient feature of *kato* showing sequences, which are analyzed next.

### *Kato* Showings

With a *kato* showing, the recipient is directed to look at something but rather than simultaneously pointing at the target, the participant producing the action brings the target closer to the recipient. Typically, the speaker approaches the recipient with the target in hand (see Kidwell and Zimmerman, 2007, p. 593). In these data, *kato* showings often involve small objects, such as berries or mushrooms, or small invertebrates such as lady bugs, which fit into the palm of one’s hand. Under such circumstances, the participant holding the object has primary sensory access to it. In our data, it is frequently the case that not only do participants direct others to look at something with a *kato* showing but they also make their own stance toward the target explicit or invite the recipient to evaluate the target. Such *kato* showings are often involved in assessing the properties or amount of the berries that have been picked, for example (see Examples 4, 6). In other words, *kato* showings make it relevant that recipients mainly look at and talk about the object of their shared attention (cf. *kato* prompts in the next section, which make also other actions such as the touching or handling of the object relevant).

**Example 4:**
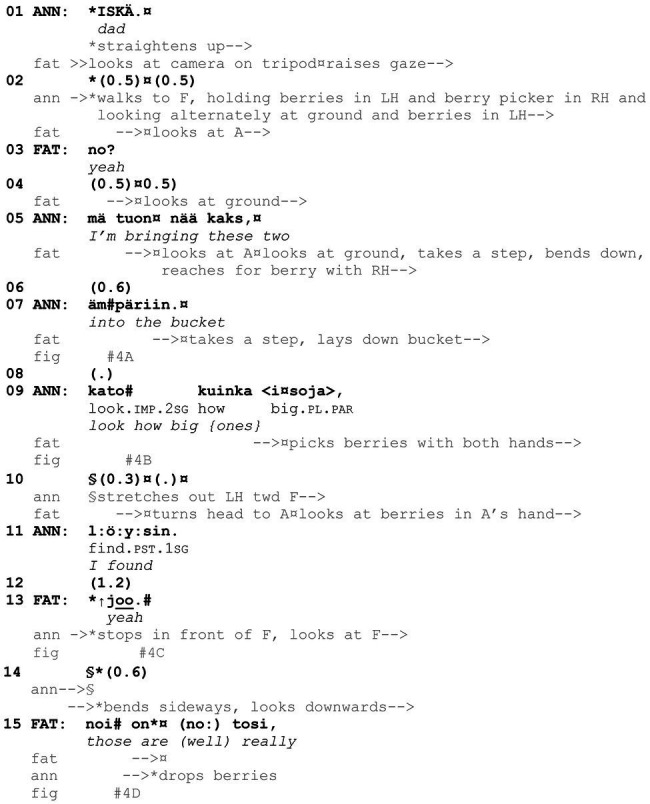
25HANS Mustikassa V (00:00:10 / 00:17:17).

**Figure 4:**
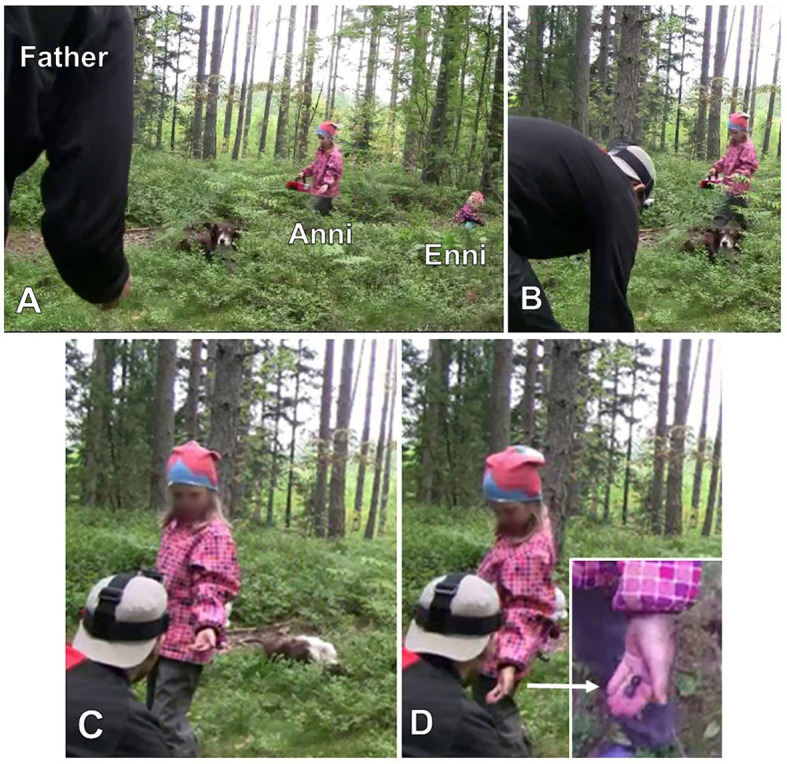
Anni walks along the path, holding the berries in her left hand; father is bending down to reach for berries with his right hand **(A)**. Anni is quite far away from father as she initiates the *kato* turn; father looks downward and orients to picking berries **(B)**. Anni stops in front of father, looking at him and holding the berries in her outstretched hand; father looks at the berries **(C)**. Anni looks downward and bends sideways to drop berries into father’s bucket; father looks at the berries **(D)**.

Example 4 presents a *kato* showing that directs the recipient to look at the target in the speaker’s hand in a particular way. The example features father and his three daughters, 6-year-old Anni, 4-year-old Enni, and 2-year-old Ella, who are picking bilberries in the woods. In the beginning of the example, they are located relatively far away from each other (see Figure 4A, Ella is not visible in the figures). Consequently, the showing action (lines 9–11) entails that Anni (speaker and shower) first moves closer to father (recipient and showee) with the target in hand.

In the beginning of the example, Anni straightens up from her picking position and breaks the silence with a loud *iskä* “dad,” thus summoning father’s attention, and then starts to walk toward him along the path (lines 1–2). After a glance and the go-ahead response *no* “yeah” from father (lines 2–4, see Sorjonen, 2001, p. 211–216; Sorjonen and Vepsäläinen, 2016, p. 246–250), Anni accounts for her coming to him, implicating that, before picking more, it is worth taking the two bilberries that she holds in her hand into father’s bucket (lines 5–7). At this point, they are still quite far from each other, and father goes on picking berries and only takes a second glance at Anni when she verbally refers to the target in her hand (*nää kaks* “these two,” line 5). To secure father’s attention to the berries, Anni next verbally directs him to look at them with *kato kuinka isoja löysin* “look how big {ones} I found,” specifying why father should pay attention to these particular berries (lines 9–11, Figure 4B). During her verbal turn, Anni continues to walk toward father and begins to stretch out her hand with the berries toward him after saying the word *isoja* “big {ones}” (line 10). Moreover, she produces the word slowly, pauses for half a second, and then finishes the turn with the word *löysin* “I found” with lengthened sounds, adjusting the turn to end when she is closer to father.

Father responds to Anni’s unfolding evaluative showing action in two ways: (1) by raising his gaze from the ground to the berries in her outstretched hand (line 10) and, as Anni stops in front of and turns her gaze to him (see Gerhardt, 2019, p. 153), (2) by acknowledging the target and agreeing with Anni’s evaluation of it with the dynamic response particle *joo* “yeah” (line 13, Figure 4C; see also Stevanovic, 2012, p. 848–485; Siitonen and Wahlberg, 2015, p. 82). Anni treats it as a sufficient next action by subsequently bending sideways a little, moving her gaze from father to his bucket, and finally dropping the berries into it (lines 14–15, Figure 4D). At the same time, father begins to assess the berries, displaying his independent access to them (line 15). He cuts off his turn when he drops his own berries onto the ground, missing the bucket, and produces a short side sequence, but then resumes and completes his assessment of Anni’s berries with the adjective phrase *tosi pulleita* “really puffy” (data not shown).

In our data, *kato* showings do not necessarily include an explicit verbal naming of the referent (e.g., berries), as the object of shared attention is frequently established through embodied actions or is otherwise evident based on the ongoing course of action. Nevertheless, *kato* showings may, as Example 4 has shown, include explicit evaluative elements or otherwise guide the recipient to see or experience the object in a particular way and thus indicate what kind of response is expected. In some cases, however, the recipient is not guided in this way, and the meaning and purpose of the *kato* showing need to be extracted by the recipient based on the context. This is the case in Example 5. In this example, 2-year-old Väinö is picking berries, with grandfather observing and guiding him. The older brother, Risto (4 years), has been at some distance, but here he comes to join them, and after picking a berry, shows his berry container, a paper cup, to grandfather. Risto’s showing turn, *kato pappa* “look grandpa” (line 13), is inserted in the middle of the collaborative, ongoing picking activity between Väinö and grandfather. The latter is assisting Väinö by holding on to his cup and by holding a branch of berries up so that Väinö can pick the berries more easily (line 1). They also jointly establish how the berries seem to be hiding in the thick vegetation (lines 1–3).

**Example 5:**
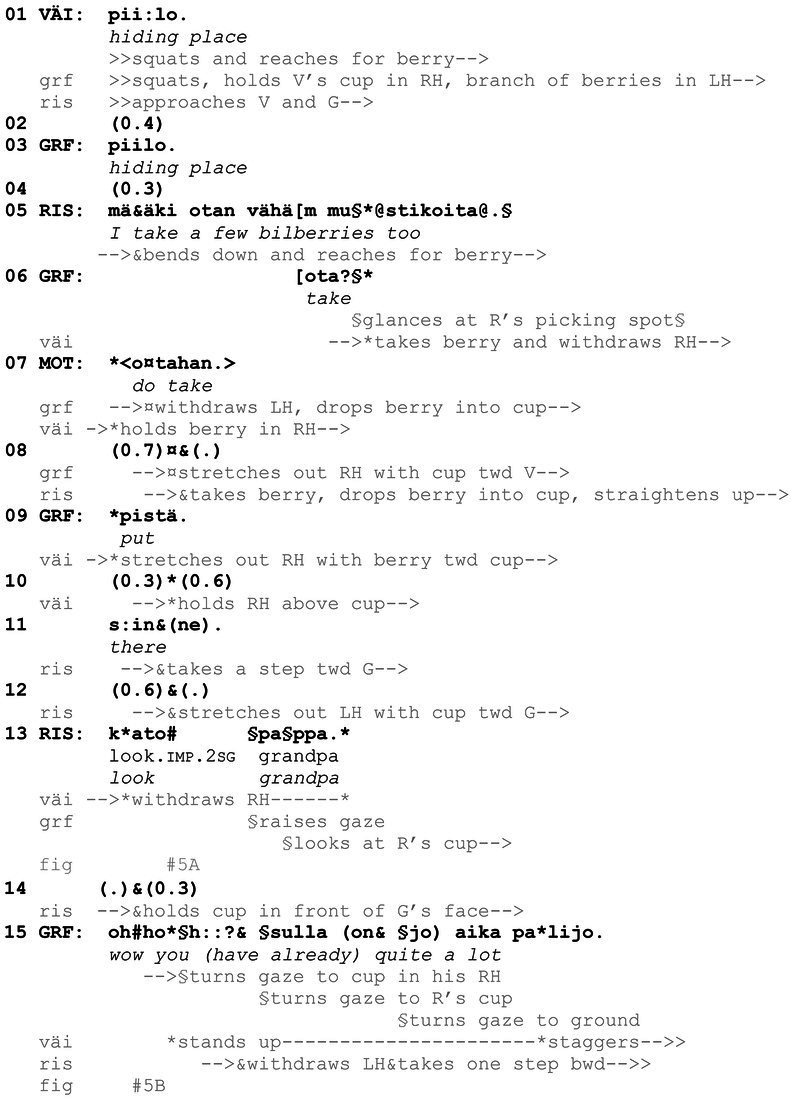
22HANS Mustikassa II (00:09:73 / 00:09:09).

**Figure 5:**
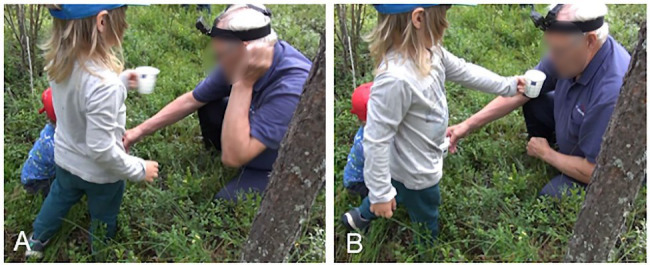
Risto stretches out his arm **(A)**. Risto holds the cup under grandfather’s face **(B)**.

As mentioned, Risto has been picking berries at some distance, but now he approaches Väinö and grandfather, and, as he gets closer, he announces his intention to join them in picking berries at the same spot (line 5). Both grandfather and mother (the latter is videorecording the situation) ratify his aim: responses designed with imperatives, here *ota* “take” (line 6) and *otahan* “do take” (line 7), may be used to encourage and support the course of action indicated in the prior turn (Sorjonen, 2017, p. 246, 268). After Risto has picked a berry and dropped it in his cup (lines 8–11) and grandfather has simultaneously guided Väinö to do the same, Risto straightens up, takes a step closer to grandfather and stretches out his hand with the cup, showing it to him (lines 11–13, Figure 5A). He also utters *kato pappa* “look grandpa,” holding the cup directly under grandfather’s face and line of sight (line 13, Figure 5B). As grandfather’s attention has been mainly on Väinö, Risto directs grandfather’s attention both with *kato* “look” and the summons *pappa* “grandpa,” in order to secure his attention on the cup. In response, grandfather produces a positive assessment of the number of berries (line 15). The relevance and purpose of the showing is thus made explicit by grandfather’s turn. Risto accepts grandfather’s response as sufficient by withdrawing the cup, and berry picking continues. It should be noted that the way in which the *kato* showing is accomplished in this example is perhaps more typical of interaction between children and adults: it is less likely that adult participants would design their showing action by taking the object so close to the recipient as here, as *kato* showings are primarily designed for looking at and talking about the object (see Example 7 for comparison with *kato* prompts, where smelling can form the next relevant action by the recipient).

**Example 6:**
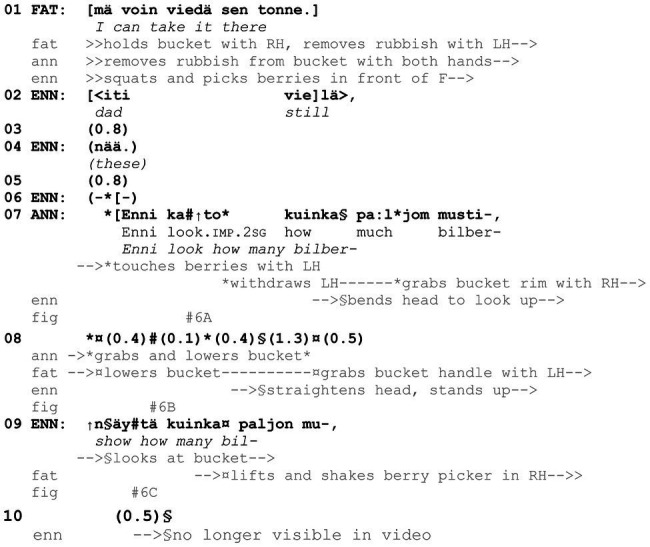
25HANS Mustikassa V (00:17:03).

**Example 7:**
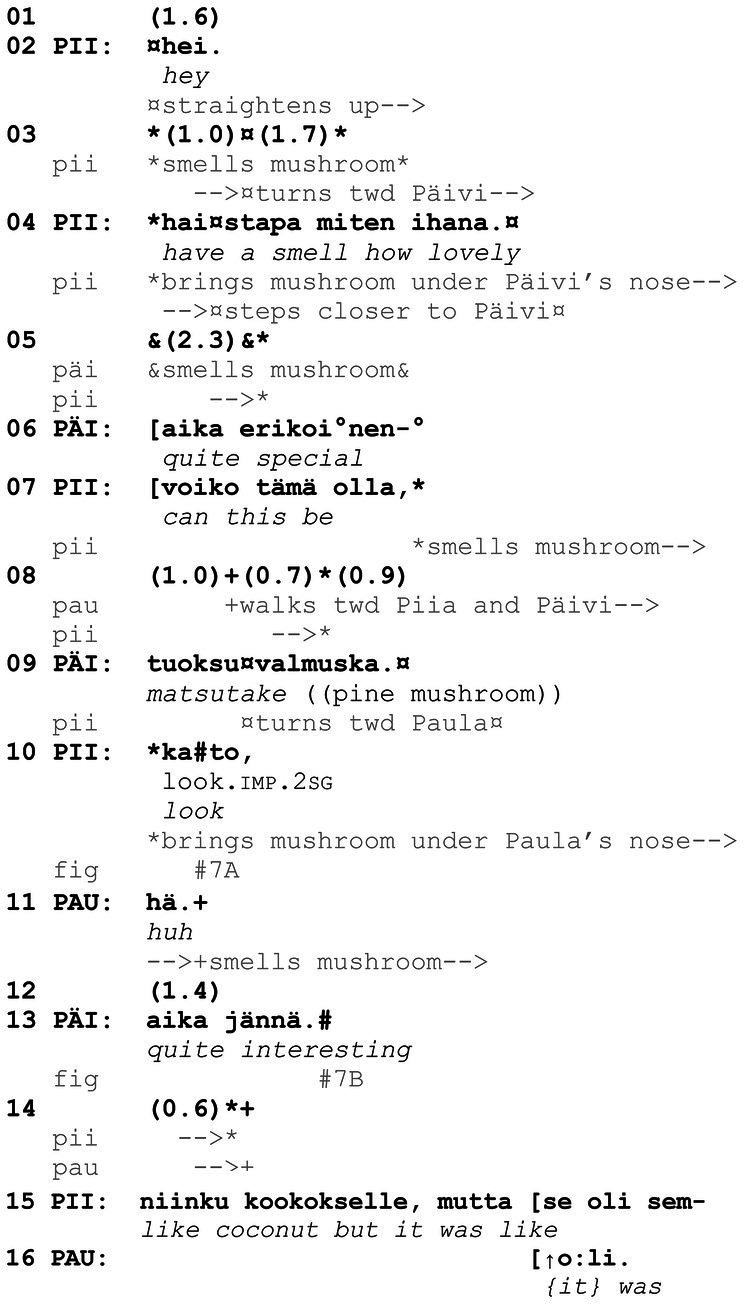
07HANS Sieniretki (00:04:14).

The importance of the material environment and the participants’ relative position to the to-be-shown object are highlighted in the next example. In contrast with Examples 4, 5, here the target of the *kato* showing, bilberries, is and needs to be explicitly named, as the bilberries become visible and thereby accessible to the recipient only after the *kato* turn. The example is from the same event as Example 4, in which father is picking berries with his three daughters. Prior to the extract, father is standing close by to Anni and Enni, emptying his berry picker into a bucket. In relation to this, Anni initiates a sequence during which father and Anni establish that the bucket is full, and Anni offers to fetch a lid to it. At the start of the extract, it can be seen that father declines the offer by stating that he can take the full bucket to where they have their other equipment (line 1). However, before father leaves, Anni invites Enni to assess the great number of berries in the bucket with a *kato* turn (line 7).

**Figure 6:**
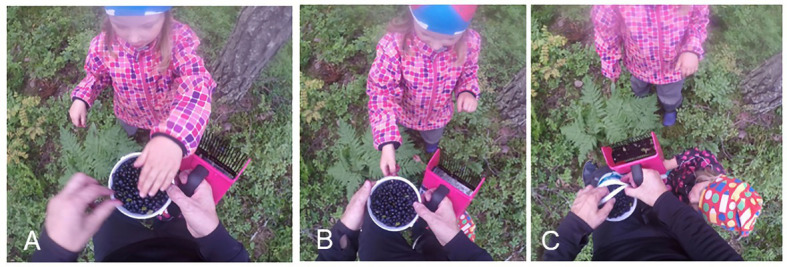
Father holds the bucket and removes pieces of rubbish; Anni touches the berries **(A)**. Anni pulls the bucket down **(B)**. Enni looks at the bucket in father’s hands **(C)**.

During the discussion between Anni and father, Anni’s younger sister Enni has been picking berries just next to father’s feet, between him and Anni. Upon hearing that father is about to go away with the bucket from their current spot, Enni produces what seems to be a request to take along the berries from her as well (lines 2, 4). Before Enni gets to put her berries in the bucket (data not shown), Anni invites her to appreciate the amount of berries already in the bucket with a *kato* showing, *Enni kato kuinka paljom musti*- “Enni look how many bil-” (line 7). Since Enni is squatting and both Anni and father are standing up, she is not able to see into the bucket (Figure 6A; Enni is not visible as she is under father’s arms and the bucket at this point). To help Enni gain visual access to the berries, which is central to the showing action as well as for being able to agree or disagree with the assessment, Anni grabs the bucket and pulls it down (Figure 6B). Father collaborates, and together they make the contents of the bucket visible to Enni (line 8). Enni has looked up during the *kato* turn and, when the bucket is brought to her eye level, she peeks into it (Figure 6C). At the same time, she requests to see the great number of berries (line 9). This verbal turn, as well as visibly looking at the berries is treated as a sufficient response to the *kato* showing by others.

Due to the specifics of the ongoing action and the participants’ involvement in it, Example 6 includes a linguistically full *kato* showing, where the recipient is guided on what the target object is and also on how it should be seen. Further, the *kato* showing is based on making a movable object accessible to the recipient jointly by more than one participant. Father mainly holds the bucket, while Anni directs its movement, and at the end father holds it in view of the recipient, Enni, with outstretched arms. The importance of the timing and synchronization of verbal, bodily, mobile, object-, and space-related resources in relation to the ongoing activity are explored further in *kato* prompts.

### *Kato* Prompts

While *kato* noticings and *kato* showings, discussed in the previous sections, direct the recipient to turn their gaze toward and look at a target, the *kato* prompts in the data direct the recipient to do something to or with the target. Vision may often be involved along with other sensory modalities, but in contexts where *kato* prompts are produced, the relevant response is for the recipient to manipulate a target that is already at hand or made available to them during the prompt. In these cases, the target of the action is typically neither mentioned explicitly, nor is the nature of the prompted action spelled out (i.e., what it is exactly that the recipient is expected to do). These can be inferred from other components of the prompting action than its linguistic design, especially from its position in the ongoing sequence.

Example 7, which is from an organized mushroom-picking excursion, is an illustrative case of *kato* prompting (an analysis of the extract is provided in Finnish in Siitonen et al., 2019, as Example 3). Piia and Päivi have each picked mushrooms and now get together to inspect their finds (on inspection of objects, see Keisanen and Rauniomaa, 2019; Mortensen and Wagner, 2019). At the beginning of the extract, Piia is squatting down with her back toward Päivi, but during the attention getter *hei* “hey” (line 2), she begins to get up and turn around. As she gets up, Piia first smells the mushroom in her hand and then directs Päivi to smell it as well, using the perceptual verb *haistaa* “to smell, to have a smell” (*haistapa miten ihana* “have a smell how lovely,” line 4). In this way, Piia invites Päivi to inspect, classify and assess the find together with her (on such sequences in foraging, see Keisanen and Rauniomaa, 2019). When Paula, who has been standing at some distance from Piia and Päivi, joins them, Piia invites her to do the same, now using *kato* “look” (line 10).

**Figure 7:**
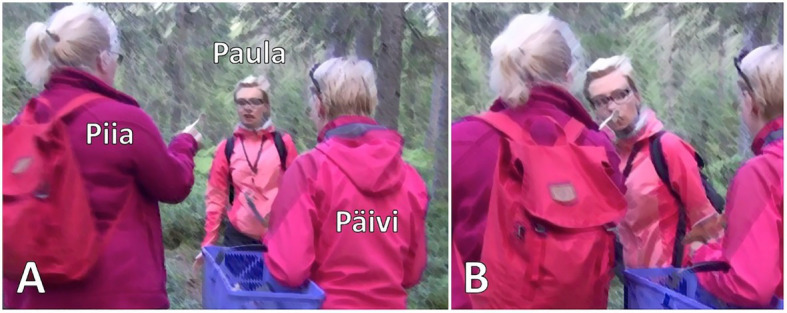
Piia brings the mushroom under Paula’s nose **(A)**. Paula smells the mushroom, her gaze away from the mushroom and co-participants **(B)**.

Piia and Päivi inspect the mushroom by smelling it and assess the smell (lines 4–6). After both of them have had a smell, Piia begins to produce a classification or identification of the mushroom, formatted as an interrogative *voiko tämä olla* “can this be” (line 7). During Piia’s utterance, Paula first directs her gaze toward Piia and Päivi and, during the pause that follows, begins to walk toward them. As Paula gets closer, Päivi provides a candidate identification of the mushroom, *tuoksuvalmuska* “matsutake” (line 9), and Piia turns toward Paula and brings the mushroom under Paula’s nose (Figure 7A). In addition to making the mushroom available to Paula in this way, Piia invites Paula to join the ongoing inspection, classification and assessment of the mushroom with the verbal turn *kato* “look” (line 10). Albeit her open repair initiator *hä* “huh” (line 11; see Haakana, 2011) displays some confusion, Paula immediately orients to smelling the mushroom as the relevant action to perform (Figure 7B). This orientation is visible not only in her leaning in slightly to smell the mushroom but also in her gaze conduct during the smelling: she turns her gaze away from the mushroom as well as from her co-participants, sideways, and unfocused (Figure 7B; on participants’ typical gaze conduct during smelling, see Mondada, 2018). That is, although the prompt includes a verb of visual perception, Paula focuses on the olfactory cues that her co-participants have also drawn on. After Paula has smelled the mushroom, Päivi and Piia continue to assess it (lines 13 and 15, respectively), and Paula produces an agreeing response that closes the sequence (line 16; see Hakulinen and Sorjonen, 2009, p. 127–128 on agreeing verb repeat responses in Finnish).

In sum, in Example 7, two participants are engaged in inspecting an object by smelling it. As soon as another participant has walked close enough, she is successfully invited to join the ongoing inspection through a prompt that consists of the verbal *kato* “look” and the bringing of the mushroom under her nose, available for smelling. What is in effect being prompted can be inferred on the basis of the activity already under way, that is, what the participant producing the prompt has just been engaged in. Similarly, in Example 8, a *kato* prompt directs the recipient to carry out an embodied action on objects that are within reach and thus to contribute to an ongoing course of action and thereby reflexively constitute it. Risto (here, 4 years) is in the woods with his mother and grandfather, picking bilberries. Grandfather is at some distance from Risto, when Risto discontinues picking berries at his current spot and starts to walk toward grandfather. As Risto gets closer, grandfather produces a *kato* prompt to direct him to continue the berry picking at this new location (line 2).

**Example 8:**
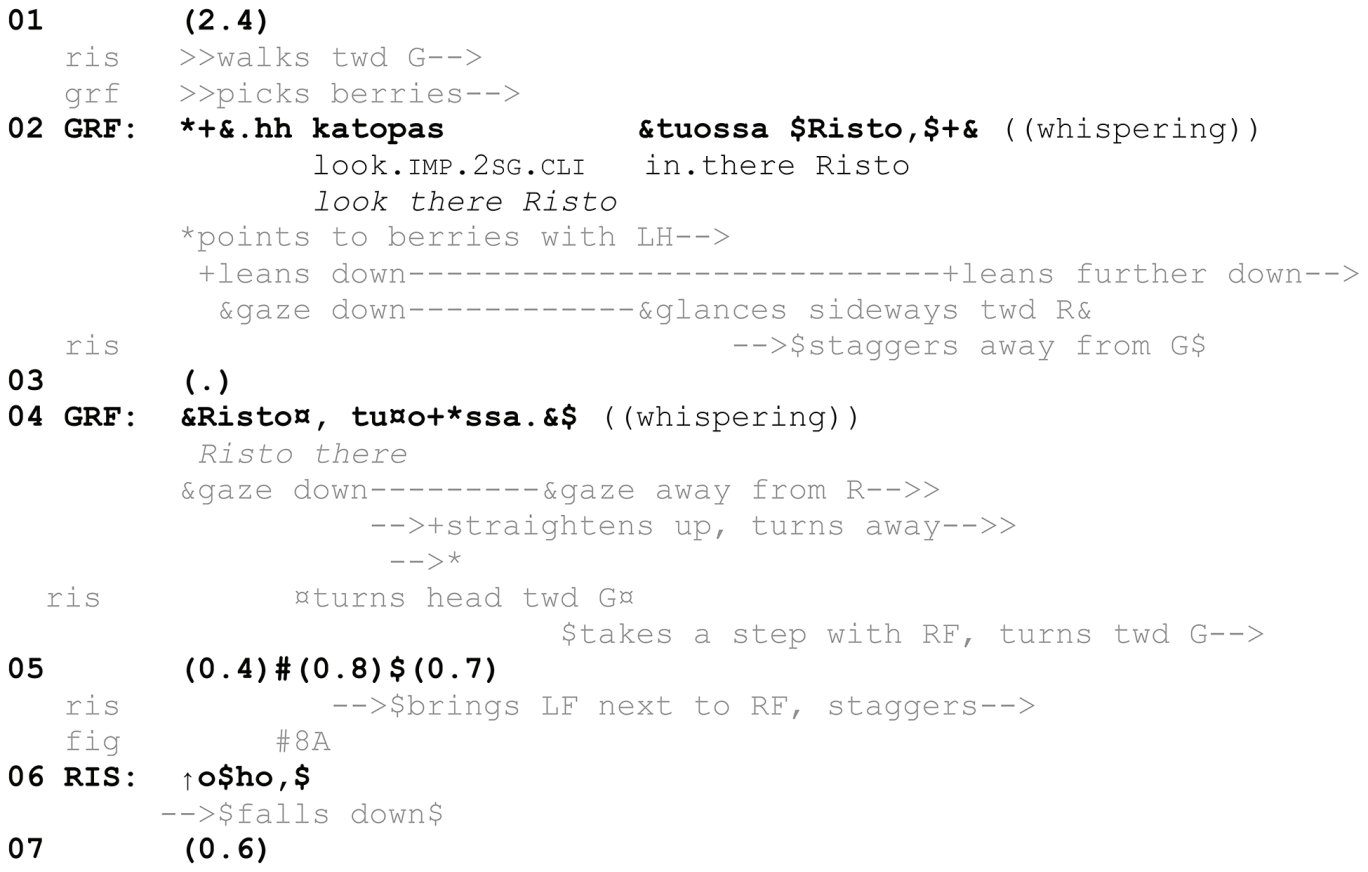
HANS06 Mustikassa (00:07:14 / 00:08:03).

Grandfather has been picking berries, but as Risto gets closer, he discontinues the picking, stretches out his left hand to point at some berries and utters *katopas tuossa Risto* “look there Risto” (line 2). The prompting action directs Risto to continue berry picking where grandfather is pointing at. The clitic -*pas* in *katopas*, in particular, indicates that it should be unproblematic for Risto to comply with the directive (on the clitic -*pas*, see Siitonen et al., 2019, p. 535; see also Carlson, 1993, p. 90). As he leans down toward the berries, grandfather glances at Risto (line 2), in time to see Risto struggling in the undergrowth and staggering away from him. Grandfather then repeats the summons, *Risto*, with prosodic emphasis, followed by a repeat of the deictic term *tuossa* “there” (line 4). At the same time, grandfather leans further down, now almost touching the berries. The repeats accommodate Risto’s staggering, which has delayed the possibility of compliance. In other words, grandfather’s repeating parts of the prompt renews its sequential implications and provides Risto another opportunity to display embodied compliance with it, encouraging him to continue picking berries at this new spot (on repetition as a means of renewing an utterance’s sequential implications, see Schegloff, 2004; see also Rauniomaa, 2008).

**Figure 8:**
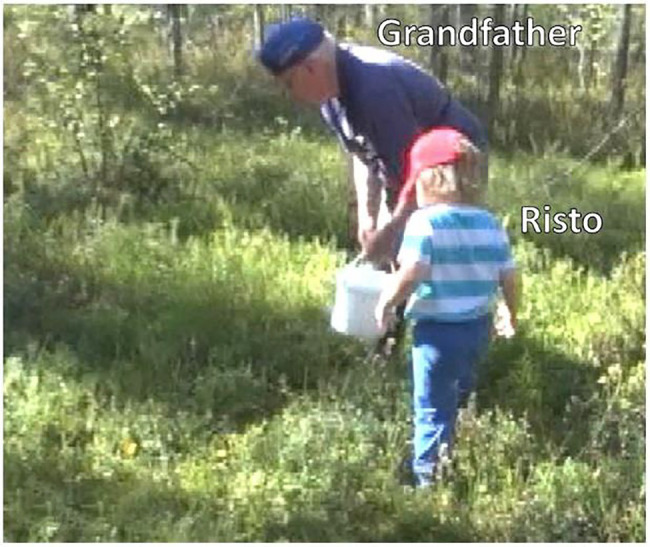
Grandfather turns away and Risto orients toward the picking spot.

As grandfather is producing the prompt, Risto arrives at his side (line 2). He slows down, takes a step with his right foot, and attempts to bring his left foot next to it, in order to position himself appropriately for the picking of berries (lines 4–5). However, he stumbles and falls back on his bottom, letting out a response cry, *oho* “oh, oops,” to indicate trouble with his movement (line 6; see Goffman, 1978 on response cries). Even though Risto does not yet start picking berries, because the final embodied compliance is interrupted by the fall, his movement up until that point projects compliance with grandfather’s directive *kato* prompts. Moreover, compliance is also projected by Risto’s gaze behavior. When Risto staggers, the staggering also causes him to turn his gaze away from grandfather (line 2). However, as he is summoned again (line 4), Risto turns his gaze toward the spot grandfather is pointing at and not, for example, toward grandfather (Figure 8). In other words, Risto’s focus of attention is appropriate in terms of the directive *kato* prompt and indicates that he is about to comply with it. As Risto has now displayed imminent compliance by his gaze direction, movement, and other embodied conduct, grandfather withdraws his hand from the point and turns away to continue picking berries nearby (Figure 8). The fact that he does so before Risto has actually started picking berries is an indication of his treating Risto’s conduct so far as projecting an appropriate response to his *kato* prompt.

Examples 7, 8 have shown that particular embodied actions, without any immediate verbal uptake, are treated as appropriate responses to *kato* prompts and that, while the embodied actions may include relevant gaze shift toward the target objects, “looking” is neither the only nor the primary response that is expected. Example 9 highlights this even further. Here, Väinö (2 years) is directed by grandfather to pick a bilberry. Grandfather has walked a little further from the others to search for bilberries and, having found some, now invites Väinö to join him (*tuuppa kattoon täältä* “come have a look here,” line 1).

**Example 9:**
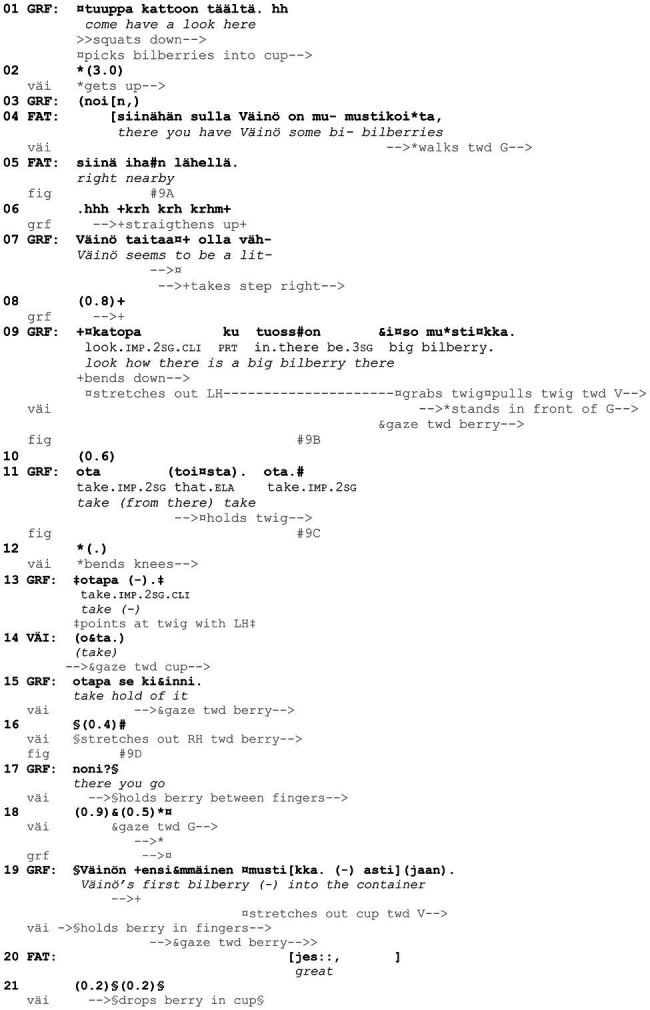
22HANS Mustikassa II (00:04:26).

**Figure 9:**
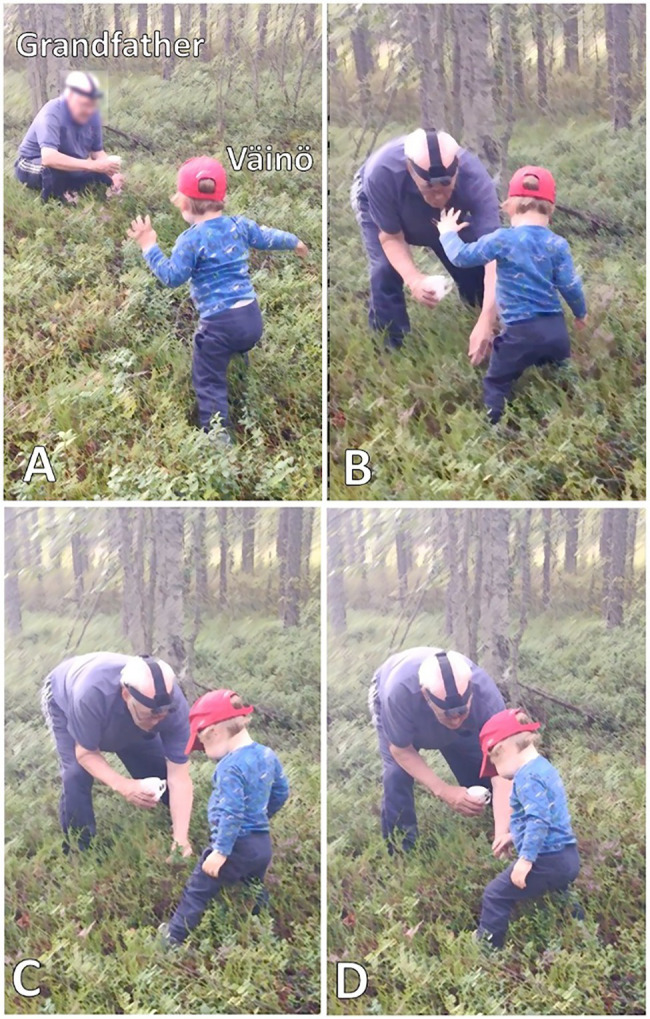
Grandfather picks bilberries and Väinö walks toward him **(A)**. Grandfather points at a berry in front of Väinö **(B)**. Grandfather holds berry twig with his left hand **(C)**. Väinö stretches out his right hand toward the berry **(D)**.

Väinö takes up grandfather’s invitation and begins to make his way through the undergrowth toward grandfather (Figure 9A). As Väinö is getting up and finding his balance, father points out that there are in effect bilberries “right nearby” where Väinö currently is (lines 4–5), which treats the ongoing activity to be about picking, rather than searching for, berries (see Keisanen et al., 2017 on iteration of the two phases in foraging). In this context, then, grandfather’s turn *katopa ku tuosson iso mustikka* “look how there is a big bilberry there” (line 9, Figure 9B), produced once Väinö has reached grandfather’s side, would serve as a directive to engage in the bodily action of picking. The evaluative naming of the referent, *iso mustikka* “a big bilberry,” would further explicate why picking is particularly worthwhile in the present spot (see Keisanen et al., 2017 on how foraging is constructed as a meaningful activity to be appreciated). However, because the referent is made explicit in this way, grandfather’s prompting *kato* turn can also be heard as a noticing, in which case “looking” would be the relevant response (see section “*Kato* noticings”).

Väinö first seems to treat grandfather’s *kato* turn as a noticing: once he stands in front of grandfather and has found his balance, he brings his hands to his sides, without in any way projecting that he would be about to pick the berry. Grandfather then employs a verb that directs Väinö’s bodily actions in a more straightforward manner, *ota* “take” (line 11, Figure 9C).^3^ Väinö first continues to stand straight with his hands on his sides but finally bends his knees slightly (line 12, Figure 9D). Väinö then repeats the word *ota* “take” (line 14), and grandfather employs it to produce one more directive, *otapa se kiinni* “take hold of it” (line 15), which is the most explicit in terms of what to do with the berry. After grandfather’s last directive, Väinö stretches out his right hand toward the berry (Figure 9D) and, after an encouraging *noni* “there you go” (line 17) by grandfather, holds the berry between his thumb and index finger and picks it from the shrub. Grandfather now frames the occasion as “Väinö’s first bilberry,” and both father and grandfather praise Väinö for the accomplishment (line 19 onward).

Examples 7–9 have shown that participants produce prompts to invite others to contribute to a course of action that is relevant for their ongoing joint activity. In Example 7, the recipient has been peripherally involved in the sharing of finds before she gets close enough to the others to be able to inspect and assess a particular mushroom. In Example 8, the recipient has just reached the others when he is prompted to continue picking berries at this new spot. Example 9 also shows how, in and through mundane family interaction, a child is socialized not only to the particular nature-related activity of foraging but also to understanding how various verbal, bodily, and spatial resources may be employed to accomplish different social actions.

## Directive *Kato* Actions as Grammar-Body Assemblages

Having discussed the different directive *kato* actions through a number of illustrative cases, we now summarize and elaborate on our findings about the sequential position, embodied design, and specific linguistic turn design features of each action.

First of all, both *kato* noticings and *kato* showings in our data direct the recipient to look at something visible in the physical space, but they differ in that they may be produced in different sequential positions and formatted with different verbal, bodily as well as object- and space-related resources. *Kato* noticings typically initiate a new sequence, which may even overlap with an ongoing course of action as the participants move from one place to another as part of their nature-related activity and adjust their actions to objects appearing in the passing world. *Kato* showings, in turn, typically refer to objects that are showable here and now because they are somehow significant for an ongoing activity that at least the participant doing the showing is engaged in. The participant producing the action, therefore, has primary access to the object. Under such circumstances, *kato* noticings usually name or categorize the target (an anthill and a mushroom), whereas *kato* showings evaluate the target in terms of its amount or properties (a lot and big) but do not necessarily name it. *Kato* prompts, by contrast, direct the recipient to do something to or with a physical target. The target or the nature of the intended action is only rarely mentioned explicitly, but they are inferable from the sequential position of the prompt. That is, *kato* prompts contribute to a course of action that has already been established as relevant for the participants’ ongoing joint activity.

All three *kato* actions direct the recipient’s embodied conduct and occasionally make relevant a verbal response. In response to *kato* noticings and *kato* showings, the recipient looks at the target and verbally acknowledges that they have visual access to it and, consequently, a shared understanding of it. For a *kato* noticing, the latter may be achieved through negotiation between the participants, whereas in producing a *kato* showing, the participant most often displays their own stance toward the target for the recipients to align with in the same fashion. *Kato* prompts do not usually elicit an immediate verbal response. Instead, the expected response to a *kato* prompt often involves that the recipient looks at the target in question and, more importantly, manipulates the target or experiences it through other sensory modalities in similar ways as other participants have already done.

Secondly, regarding specific bodily resources, *kato* noticings are often accompanied with pointing gestures, but the participants remain at some distance from the target. *Kato* showings, in turn, often involve the participant producing the action approaching the recipient with the target in hand. In *kato* noticings and *kato* showings, both gaze and body orientation are employed to mark the location of the target and gaze is also used to monitor that the recipient directs their attention to the relevant target. Moreover, participants’ movement in space and positions in relation to the target as well as to one another are drawn on to time the actions appropriately for the recipient to be able to carry out the nominated or implied action. In *kato* showings and *kato* prompts, especially, the proximity of the target is relevant: both *kato* actions and relevant responses to them are produced only when the recipient is close enough to the target so that the target can be established or maintained as the focus of the participants’ joint attention and activity.

Finally, with regard to the linguistic design of the *kato* actions in our data, the imperative verb *kato* is involved in two distinct turn design practices. In the first, in *kato* noticings and *kato* showings, *kato* is used as a transitive verb that has the concrete meaning of “looking” and syntactically takes an object-NP or a clausal object, and, in *kato* prompts, which typically do not name the target, as a verb that is accompanied with a deictic term in a dynamic locative case (on possible objects with *katsoa* “to look,” see also Hakulinen and Seppänen, 1992, p. 530; Hakulinen et al., 2004, § 461). More specifically, should the *kato* imperative take an object-NP, it is in the partitive case^4^ and typically marked as determined with a demonstrative pronoun (see Table 2). Albeit fairly rare in our data, this was the case in Example 3, in which the speaker directed the other participants to “look *at* that lakeside” [*katto-kaa tuo-ta … ranta-a* (look.imp-2pl that-par lakeside-par)]. Should the *kato* imperative take a clausal object that explains what is relevant to look at, it is marked as an explanation or an account with a prefacing complementizer, usually *ku* “as, how,” *miten* “how” or *kuinka* “how” (see also Hakulinen and Seppänen, 1992, p. 530, Raevaara, 2011, p. 560). The complementizer *kuinka* “how” is employed especially in *kato* showings that typically display the speaker’s stance toward the target (see Table 2; see also Examples 4, 6). With regard to *kato* prompts, in turn, the *kato* imperative is accompanied with the deictic terms indicating motion to a location (i.e., where to look), *tänne* “to.here,” *tonne* “to.there,” and *siihen* “to.there,” in our data (see Table 2).

**Table 2:** Examples of linguistic turn designs in *kato* noticings, showings, and prompts.

*Kato* action	*kato* + syntactic object or deictic term in dynamic locative case	*kato* + explanation
***kato* noticing** Pointing, gaze and body orientation to target, some distance to the target Initiates new course of action	***kato* + object-NP (partitive case)** *kattokaa tuota vittu rantaa* “look at that fuck lakeside” *katopa tuota. Risto se on muurahaisen silta siellä* “look at that. Risto it is an ant’s bridge there” ***kato* + clausal object** *katopa ku Risto tääl on nuita puolukoitaki mutta eivät ole vielä kypsiä* “look Risto how there are also those lingonberries here but {they} are not ripe yet” *kato miten on puu kaatunu tonne päälle* “look how a tree has fallen down there over”	***kato* + NP explanation (nominative case in singular)** *kato muulahaitpetä* “look an anthill” *kato muurahaisen keko* “look an ant’s hill” *hei kato mutta muttikka* “hey look a bwack bwuebewwy” ***kato* + clausal explanation** *katoppa tääl on toinenki sieni* “look there is another mushroom here” *kattokaa tääl on koiran jälkiä* “look there are dog tracks here”
***kato* showing** Holding or touching target, gaze and body orientation to target, touching target Initiates evaluative course of action	***kato* + object-NP (partitive case)** *kattokaa sitä väritystä siinä* “look at the color there” ***kato* + clausal object** *äiti kato kuinka paljo meikä on saanu jo* “mother look how many {berries} I have already”	***kato* + NP explanation (nominative case in singular)** None^1^ ***kato* + clausal explanation** *itä kato. tää on (aivan) hattun muotone* “dad look. this has a (totally) funny shape” *kato nyt on etana siellä* “look now there is a slug there”
***kato* prompt** Pointing or touching target, gaze and body orientation to target, close to target Maintains ongoing course of action	***kato* + deictic term in dynamic locative case** *no kato Väinö tänne* “now look Väinö to.here” *katopa Risto siihe* “look Risto to.there”	***kato +* deictic term in static locative case** *hei kato täällä* “hey look at.here” *katopa tossa* “look in.there” *katopa tässä* “look in.here”

1 The only *kato* showing with a NP explanation in our data is *(iskä) kato partaa* “(dad) look a piece of beard,” in which the explanation what to look at is exceptionally in the partitive case because the noun *parta* “beard” is used as a mass noun or noncount noun. The recipient is shown a piece of beard lichen, which is described as “beard.” That is, the participant producing the showing is not directing the recipient to look at a particular beard [cf. *kato (tuota) partaa* “look at the beard”]. If a count noun was used to refer to the target (as an NP explanation, *kato parta* “look a beard”), the noun would be in the nominative case like in *kato* noticings (e.g., *kato etana* “look a slug”). This implies that *kato* showings do not typically introduce targets as such to the recipient, but rather introduce the target and invite the recipient to evaluate it.

In the second linguistic turn design practice, which is the more frequent one in *kato* noticings and *kato* prompts but the less frequent in *kato* showings in our data, *kato* is used as an imperative verb that has the concrete meaning of “looking” but it neither takes a syntactic object, nor is it supplemented with a deictic term in a dynamic locative case. In *kato* noticings, in particular, the speaker designs the linguistic turn by employing *kato* and a (singular) NP in the nominative case, without a demonstrative pronoun, so that the NP is not syntactically an object-NP of the verb but functions as an explanation of what is relevant to look at (see Table 2; for similar observations, see Hakulinen et al., 2003, p. 199; Goodwin and Goodwin, 2012, p. 268; San Roque et al., 2018, p. 385). Nevertheless, these kinds of utterances are frequently produced as one prosodic unit in our data. In a similar vein, in both *kato* noticings and *kato* showings, the speaker may use a clausal explanation of what to look at even though the explanation is not syntactically marked as a clausal object of the verb *kato* with any complementizer. Moreover, a typical linguistic resource used in such explanations (especially in *kato* noticings) makes use of the Finnish existential structure that establishes a containment relation between space and target so that the target is presented as a new element in the space (see Table 2, Example 2, Siitonen et al., 2019, p. 527; on the Finnish existential structure, see, e.g., Huumo, 1996). With regard to *kato* prompts, which typically do not name the target, the deictic explanation of where the target is located is indicated with a deictic term in a static locative case, *tässä* “in.here,” *täällä* “at.here,” *tuossa* “in.there,” *tuolla* “at.there,” *siinä* “in.there,” and *siellä* “at.there” (see Table 2). The internal locative case (i.e., inessive) marks the location more figure-like and the external locative case (i.e., adessive) marks the location more ground-like (Laury, 1997, p. 145).

The difference between the two linguistic turn design practices, (1) *kato* + syntactic object/deictic term in dynamic locative case and (2) *kato* + explanation/deictic term in static locative case, indicates that *kato* has lost some of its verb-like features in the latter. In that regard, its usage is getting closer in the continuum to the usage of the particle *kato,* an attention getter that does not direct the recipient to look at anything visible but, by prefacing an abstract (non-visible) explanation, rather directs the recipient to understand (Hakulinen and Seppänen, 1992). Even when the *kato* verb does not take a syntactic object, it agrees in number with the number of recipients (i.e., singular *kato* vs. plural *katto-kaa* “look.imp-2pl”), and the turn directs the recipients’ *visual* attention, in particular. As for *kato* prompts, the verb *kato* addresses the second person and bears the meaning of “looking” or “becoming aware of” (see Hakulinen and Seppänen, 1992, p. 547), but additionally and importantly, directs the recipient to carry out some embodied action other than only looking at the target. In our data, the practice of using *kato* + explanation (NP or clausal) is more frequent in *kato* noticings and *kato* prompts, whereas the practice of using *kato* + syntactic object (NP or clausal) is more frequent in *kato* showings. This makes sense because *kato* noticings and *kato* prompts typically direct the recipient to direct their attention to an object in or feature of the environment but not necessarily to engage in an intensive visual experiencing of it for an extended period of time, which may be needed in order for the participants to be able to assess the object together.

## Conclusion

In this study, we have focused on the use of a particular grammatical construction, second-person imperative of the Finnish verb *katsoa* “to look,” in carrying out three different social actions during various nature-related activities. The study has shed new light on the linguistic item *kato* “look” and furthered understandings of how participants use such imperatively formatted verbs of perception, as parts of complex, flexible grammar-body assemblages, to establish and maintain joint attention in a material world populated with physical objects. Joint attention is key to any social activity: to carry out meaningful social actions, participants rely on each other’s publicly displayed orientations and understandings and continuously update these as a particular sequence of action unfolds. Employing a methodology that genuinely focuses on interaction, then, allows us to explore “attention” not as a cognitive phenomenon that resides in the mind of an individual but as a fundamentally social process that participants of interaction accomplish together in the moment.

In contexts such as those presented in this study, where participants were engaged in activities that often required movement from one place to another and that might involve the manipulation of objects, participants frequently directed others to look at a target or to do something with or to it. That is, the forest setting itself allowed for changing sceneries with varying sources for noticings – things to admire, wonder at, or be shocked at together. Furthermore, the activities that participants were engaged in continuously provided grounds for showings, such as the qualities and quantities of finds in foraging that participants might evaluate together. Similarly, in the forest setting, prompts worked as means of inviting others to participate in, and possibly socializing the less experienced into, relevant nature-related activities.

The social actions of noticing, showing, and prompting can be seen to direct co-participants’ conduct in different ways. Noticings are treated as establishing joint visual attention to the target and inducing further talk about it, for instance, in the form of elaborations and evaluations. Showings are also treated as establishing joint visual attention to the target, but they are understood as specifically inviting a stance similar to that displayed by the participant who produced the showing. By contrast, prompts are treated as making relevant an embodied response that may involve gaze shift toward the target but, first and foremost, includes some form of manipulation or sensory experiencing of it. In this way, the different resources complement and mutually elaborate each other: it is through the particular grammar-body assemblages that the import of the actions is displayed and negotiated.

## Data Availability Statement

The datasets presented in this article are not readily available due to ethical and privacy restrictions. On giving their informed consent to be videorecorded, participants have agreed that extracts of the data may be presented in the form of transcripts and anonymized framegrabs in scholarly publications by members of the research team but that the data cannot be made publicly available in any other form. Inquiries and requests to access the datasets should be directed to TK (tiina.keisanen@oulu.fi).

## Ethics Statement

Ethical review and approval was not required for the study on human participants in accordance with the local legislation and institutional requirements. Written informed consent was obtained from the individual(s) and minor(s)’ legal guardian/next of kin, for the publication of any potentially identifiable images or data included in this article.

## Author Contributions

All authors listed have made a substantial, direct and intellectual contribution to the work, and approved it for publication.

### Conflict of Interest

The authors declare that the research was conducted in the absence of any commercial or financial relationships that could be construed as a potential conflict of interest.

## Footnotes

^1^While we have enough cases from interactions between adult participants to argue for the generalizability of our findings, many of those video recordings provide only a partial view to the actions of individual participants and make it difficult to produce detailed transcripts and truly illustrative framegrabs.

^2^Four-year-old Risto refers to the target with the phrase *muurahaisen keko* (“ant.gen hill”). Although the phrase is totally intelligible, the lexically standard expression would be the compound word *muurahaiskeko* (“anthill”).

^3^It is worth noting that, in our data, imperative forms of the verb *ottaa* “to take” are relatively frequent in directive actions in general, but they are not necessarily used in the context of foraging. What is more, when the verb *poimia* “to pick” is used in directive actions, it typically occurs in an infinitive form together with another lexical verb or a modal verb (e.g., *meehän poimiin* “go pick,” *sää voit poimia ne kaikki siitä* “you can pick them all from there”). In our entire collection of directive actions in the data, there is only one case in which the verb is used in second-person imperative (*poimipa mustikoita* “pick.cli bilberries”).

^4^Since the clausal aspect of (*kato*) noticings and showings that direct the recipient to look at the target is imperfective, the grammatical category of an object-NP is partitive (see, e.g., Hakulinen et al., 2004, § 930). Were the object-NP here in the nominative case, it would make the clause ungrammatical due to its perfective aspect [cf. *kat(s)o elokuva* (look.imp.2sg movie.nom) “watch a/the movie,” in which the clausal aspect is perfective, implying watching of the movie from beginning to end].
